# Fresh or frozen? Classifying ‘spare’ embryos for donation to human embryonic stem cell research

**DOI:** 10.1016/j.socscimed.2010.09.045

**Published:** 2010-12

**Authors:** Kathryn Ehrich, Clare Williams, Bobbie Farsides

**Affiliations:** a King’s College London, UK; b Brighton & Sussex Medical School, UK

**Keywords:** UK, Stem cell research, Embryo, Classification, Ethnography, Spare embryo, Moral work object, Built moral environment, Ethics

## Abstract

United Kingdom (UK) funding to build human embryonic stem cell (hESC) derivation labs within assisted conception units (ACU) was intended to facilitate the ‘In-vitro fertilisation (IVF)-stem cell interface’, including the flow of *fresh* ‘spare’ embryos to stem cell labs. However, in the three sites reported on here, which received this funding, most of the embryos used for hESC research came from long term cryopreservation storage and/or outside clinics. In this paper we explore some of the clinical, technical, social and ethical factors that might help to explain this situation. We report from our qualitative study of the ethical frameworks for approaching women/couples for donation of embryos to stem cell research. Members of staff took part in 44 interviews and six ethics discussion groups held at our study sites between February 2008 and October 2009. We focus here on their articulations of social and ethical, as well as scientific, dimensions in the contingent classification of ‘spare’ embryos, entailing uncertainty, fluidity and naturalisation in classifying work. Social and ethical factors include acknowledging and responding to uncertainty in classifying embryos; retaining ‘fluidity’ in the grading system to give embryos ‘every chance’; tensions between standardisation and variation in enacting a ‘fair’ grading system; enhancement of patient choice and control, and prevention of regret; and incorporation of patients’ values in construction of ethically acceptable embryo ‘spareness’ (‘frozen’ embryos, and embryos determined through preimplantation genetic diagnosis (PGD) to be genetically ‘affected’). We argue that the success of the ‘built moral environment’ of ACU with adjoining stem cell laboratories building projects intended to facilitate the ‘IVF-stem cell interface’ may depend not only on architecture, but also on the part such social and ethical factors play in configuration of embryos as particular kinds of moral work objects.

## Introduction

The meaning and value of human embryos in the associated worlds of assisted reproduction and human embryonic stem cell (hESC) research has been addressed in a growing body of social science literature (e.g., Franklin, 2006; Franklin & Roberts, 2006; Haimes & Taylor, 2009; Konrad, 2004; Morgan, 2003; Spallone, 1989; Svendsen & Koch, 2008; Wainwright, Williams, Michael, Farsides, & Cribb, 2006; Waldby, 2002; Williams, Kitzinger, & Henderson, 2003). We have contributed to this literature by exploring meanings of embryos in hESC research laboratories, assisted conception units (ACU), and laboratories performing preimplantation genetic diagnosis (PGD) (Ehrich, Williams, Farsides, Sandall, & Scott, 2007; Ehrich, Williams, Scott, Sandall, & Farsides, 2006; Williams, Wainwright, Ehrich, & Michael, 2008; Ehrich, Williams, and Farsides (2010)). We have argued that the constitution of embryos as *social objects* (Mead, 1934) is emergent and contextually contingent (Casper, 1998) and defined a further category of *moral work object* as a social object around which people make (and continually remake) meaning and organise work practices in a morally contested field (Ehrich, Williams, & Farsides, 2008).

In this paper we build on these concepts to explore how staff in the related ‘social worlds’ (Strauss, 1978) or ‘communities of practice’ (Lave & Wenger, 1991), of assisted conception, PGD and hESC research in the United Kingdom (UK) continually ‘remake’ the negotiated category of ethically acceptable ‘spare’ embryos. We analyse professional views, practices and transformations of meaning entailed in constituting embryos as socially and ethically suitable for transfer from the ‘pregnancy trail’ (Cussins, 1996) to the ‘research trail’ (Parry, 2006). Our analysis supports the work of Svendsen and Koch (2008), who investigated clinical and social practices in a Denmark clinic that transformed the meaning of embryos from that of potential babies to ‘spare’ embryos that could legitimately be recruited for creating potential stem cell lines; and Franklin’s (2006) position that the capacity of embryos that were formed in the context of reproductive labour (Cussins, 1996; Thompson, 2005) to transform into colonies of regenerative cells gives them a ‘dual reproductive identity’.

Central to these processes is the articulation of clinical, technical, social and ethical contingencies in classification work that allows transformations of embryos in both senses. For this paper we draw on Bowker and Star’s (1999) analysis of how classification systems (e.g., in medical, scientific and racial classification) can be read as political and cultural productions that function as clues to embedded forms of knowledge. All categories privilege some points of view and make others invisible. Embedded knowledge may ordinarily be invisible unless the classifications become objects of contention. In the clinics participating in our study, decisions about the fate of embryos incorporated a great deal of nuanced, complex knowledge and communication with clients that were not recorded explicitly as part of the classification of embryos, but are of interest because the category of ‘spare’ embryo remains contentious.

Classification processes also produce various kinds of infrastructure (e.g., the internet or medical diagnosic systems). Bowker and Star draw on the idea of material technical artefacts (e.g., bridges, buildings, roads) embodying social and political relations (Winner, 1980) in formulating their concept of “the architecture of classification schemes” which they argue “is simultaneously a moral and informatics one” (1999: 324). They have illustrated the idea of the ‘built moral environment’ (Bowker & Star, 1999; Star, 1999) primarily in terms of information infrastructures which incorporate both material and conceptual architecture. Here we use the concept to investigate the link between classifying, or configuring embryos as particular kinds of moral work objects and effects of this work of significance to the architectural ‘built moral environment’ of linked ACU and stem cell laboratories. Embryo classifying practices are studied here to access social, cultural and ethical elements of the story of the built environment of linked ACU and stem cell laboratories in a similar way to how “a city planner or urban historian would leaf back through highway permits and zoning decisions to tell a city’s story” (Bowker & Star, 1999: 2–5).

The ‘contested categories’ used to determine the fate of in-vitro fertilisation (IVF) and PGD embryos are historically and locally contingent, and never ‘merely’ technical: there is “a constant interplay between contestation and stabilization of categories, as these are negotiated and reconfigured” (Bauer & Wahlberg, 2009: 4). We are particularly interested in the effects of social and ethical aspects of embryo classification work, and the fluidity and naturalisation (or contestation and stabilization) of these classifications at the IVF-stem cell interface. *Fluidity* allows for movement within classification systems and room for variation in practice to accommodate uncertainty, the rapidly shifting science and policy environment, and socially and ethically contested aspects of particular categories. *Naturalisation* describes a phase when some of the complexities of everyday categories become possible to forget or ignore, and certain aspects of classification work become more fixed or stable, enabling their more taken for granted use and thus facilitating some routine work in particular communities of practice. These concepts will be used to analyse how categories to describe ‘spare’ embryos (e.g., unused ‘frozen’ embryos, and embryos determined through PGD to be genetically ‘affected’) become socially and ethically acceptable types of embryo to donate for hESC research.

## Background

Following the UK Stem Cell Initiative that funded the building of the UK Stem Cell Bank, the Medical Research Council (MRC) “provided a further £2 m to seven consortia linking IVF clinics and stem cell researchers to address the bottleneck in accessing high quality surplus embryos for stem cell research, and [in 2004] £1.2 m to support the upgrades of five IVF clinics to comply with current good manufacturing practice (GMP) standards for derivation of clinical-grade embryonic stem cells lines suitable for therapeutic applications (jointly with the Department of Health)” (MRC, 2008, pp. 4–5). The new therapeutic grade hESC derivation laboratories to be built within ACU were intended to facilitate the ‘IVF-stem cell interface’, that is the flow of ‘spare’ embryos to stem cell laboratories (Franklin, 2006, MRC, 2008). This was partly to avoid damage or contamination of fresh embryos in transport from the ACU to the stem cell laboratory, and because fresh embryos were thought at that time to be more successful at creating hESCs than frozen ones. Embryos continue to be regarded as a precious resource that should not be wasted, but there are tensions between the view that they are “rarely truly surplus” in assisted reproduction (Brison & Lieberman, 2003), and on the other hand the position taken by the House of Lords Committee on Stem Cell Research that “embryos should not be created specifically for research purposes unless there is a demonstrable and exceptional need which cannot be met by the use of surplus embryos” (POST, 2002, p. 3). The vast majority of hESC research carried out in the UK following implementation of the 1990 HFE Act uses ‘spare’ IVF embryos, with only 5% created specifically for research in 2006/2007 (HFEA, 2007).

Extensive literature exists regarding the moral status of embryos and their use in research (e.g., Mulkay, 1997; Ehrich et al., 2008); and on issues for *donors or potential donors* of embryos for research, e.g., religious and cultural aspects (Barratt, St.John, & Afnan, 2004); ethical and policy issues (Cohen, 2009); attitudes and decision-making (de Lacey, 2006, 2007); and informed consent (Heng, 2006). McLeod & Baylis argue vehemently that IVF patients should not be asked to donate their fresh embryos to stem cell research because it is in their interests to have “time and distance from their IVF treatment that would allow them to reflect carefully” (2007, p. 477). Haimes and Taylor investigate the extent to which IVF patients understand “how the quality of embryos was judged and how this grading affected decisions about freezing or use in research” (2009, p. 2144). The clinic they studied operated a policy (at that time) of only freezing embryos if there were at least four top quality embryos left over after one or two were transferred in the fresh cycle, raising the issue of ‘the troubling third embryos’ (top quality but ‘spare’).

However, with the exception of Svendsen and Koch’s (2008) Denmark study, there is a lack of empirical social science or ethics research on the views of *professional staff* and in particular, the social and ethical aspects of the *classification* of ‘spare’ embryos for donation to research. We see this paper as complementary to Svendson & Koch’s study and Haimes and Taylor’s “opening up for discussion the ‘black box’ of the provision of embryos” (2009, p. 2149), because we provide data on how social and ethical issues play an intrinsic part of the classification process. The broad acceptance for *professional staff* of using ‘spare’ embryos rests on a number of contingent judgments. By comparison to the literature on social and ethical issues for *donors*, these embedded judgments are not often articulated and to our knowledge are rarely reported. This paper addresses that gap, adding to the small but growing body of ethnographic research on the views and experiences of *staff* working in reproductive health technologies in the UK (Ehrich et al., 2006, 2007, 2008; Franklin & Roberts, 2006; Roberts & Throsby, 2008).

## Methods

We report on one aspect of a multidisciplinary, ethnographic study that explores the views, values and practices, or ethical frameworks, drawn on by professional staff in three linked ACU and stem cell laboratories in relation to embryo donation for research purposes, particularly for hESC research. The three sites in our study had been awarded grants to become single-facility IVF and hESC derivation centres. Following national and local Research Ethics Committee approvals, the study methods include clinic and laboratory observation, interviews and ethics discussion groups (EDGs) (Alderson, Farsides, & Williams, 2002) with staff from ACU and linked stem cell laboratories in the UK. Staff disciplines include nursing, obstetrics and gynaecology, embryology, stem cell scientists, counselling, and clinical and science management. As a multidisciplinary team comprised of three social scientists, an ethicist, and a consultant embryologist/ACU director, together with collaborators with clinical and legal expertise, we explore the social processes, meanings and institutions that frame and produce ‘ethics’ and ‘ethical problems’ in these settings.

The study sites are three ACU in teaching hospitals in England, which offer a mixture of National Health Service (NHS), privately, or ‘self funded’ NHS treatment, and three stem cell laboratories at the universities associated with these hospitals. The clinics provide a range of services including IVF to women and couples who need fertility treatment.

Participants from across a broad range of disciplines were recruited by group introductions to the study, followed by individual approaches from the main researcher (KE). The interviews were conducted by the main researcher as ‘guided conversations’ (Lofland & Lofland, 1984), lasting between one and 2 h. Open-ended questions and an informal interview schedule were used, including topics such as the ethical differences between donation of fresh and frozen embryos; and how embryos are selected for treatment, cryopreservation or disposal.

For our analysis of the transcripts we used a modified version of the framework approach (Ritchie & Spencer, 1994) following close readings of the transcripts. Emerging themes from the study as a whole were discussed in the team meetings. While we do not claim that the quotes we present here are statistically representative of the views of staff in our sites, or in the UK generally, they illustrate common themes that arose from the fieldwork at these sites. For this paper, because classification is mainly the work of embryologists, we use more quotes from that group than, for example, stem cell scientists. However, classification work necessarily takes place within wider and inter-related communities of practice, which we hope will be apparent.

We note that for this paper we use the terms ‘women/couples’, ‘embryo donors’, and ‘patients’ to refer to people receiving treatment from the ACU and PGD service; and ‘participants’ to describe the members of staff who engaged in interviews and EDGs. We refer to ‘staff’ in general to include staff from professional groups such as nursing, stem cell science, embryology, obstetrics and gynaecology, but refer to more specific categories of staff when appropriate. We acknowledge that for all of these practices some readers might prefer other conventions. We also emphasise that in such a rapidly developing field, technologies may appear to be more developed at certain sites as an effect of the timing of our fieldwork, which spanned a period of 21 months. Finally, we note that embryos can be donated for a range of research projects, e.g., to improve assisted conception techniques, but we focus in this paper on hESC research.

## Results

We interviewed 44 members of staff and held six ethics discussion groups at our study sites between February 2008 and October 2009. In two of the sites the number of *fresh* IVF embryos donated through the ACU for research in the linked hESC laboratories was negligible. In the remaining site, only ‘affected PGD embryos’ (i.e., those embryos that had tested positive for a known genetic condition) were being donated in the fresh cycle to the newly built hESC derivation laboratory. An important finding was that many of the ACU and hESC research staff said they were more ‘comfortable’ with donation of frozen, not fresh, embryos, to hESC research.

This was an unanticipated finding, as we had expected from the decision to build linked ACU and hESC derivation laboratories that donation of fresh embryos to research would become standard practice. We were aware that policies on grading and freezing varied in different units, but understood that the category ‘spare fresh embryo’ was sufficiently stable to expect fresh embryos to be donated for research on a widespread basis. We were therefore interested to know why donation of fresh embryos to hESC was not regular practice at our three study sites.

One explanation was that the new ACU located laboratories had only recently become fully operational, and at one site there was a temporary lull in pursuing stem cell research. However, the resolution of these factors did not appear likely to change the situation we focus on in this paper. Rather, we found indications to the contrary in our participants’ articulation of values and ethical views embedded in descriptions of the treatment process and classification of embryos.

Our analysis is organised into themes to include several factors that might help to explain the overall finding of fresh embryos not being used for hESC research at these sites. Each theme corresponds to embedded, inter-related clinical, social, ethical and technical contingencies in the classification and selection of embryos for treatment that influence and underpin decisions about possible donation of fresh or frozen embryos to research. Social factors articulated by staff included aspects of couples’ histories, religious beliefs and economic considerations, but we focus specifically here on those that relate to the classification of embryos. These include acknowledging and responding to uncertainty in classifying embryos; operating a ‘fluid’ grading system to give embryos ‘every chance’ of staying on the pregnancy trail; tensions between standardisation and variation in enacting a ‘fair’ grading system; enhancement of patient choice and control, and prevention of regret; ethical preferences for grading blastocyst embryos; and ethical preferences for seeking donation of frozen and PGD embryos.

## Themes

### Uncertainty in classifying embryos to transfer

Uncertainty is a recurring characteristic of the work done in this setting:

‘When you get a failed fertilisation, the first thing patients will say, ‘Well why did it happen?’ And you have to say, ‘Well really we don’t know.’ We can say that the eggs haven’t bound, the sperm hasn’t bound to the eggs or the eggs haven’t let the sperm in, but we don’t know why it’s happened. And it’s the same with embryos implanting, you know, they’ll say, ‘But you said we had grade one embryos, why haven’t they implanted?’ Again you don’t know. ‘ (Embryologist 7)

Embryological and clinical assessment of the suitability of embryos for infertility treatment or for PGD takes into account factors such as the number, shape and regularity of the cells, and developmental progress for each day post-fertilisation. One or two embryos (sometimes three for women over 40) are selected as the ‘best’ ones to transfer, using a system of five grades. Any embryos that are not transferred in the fresh cycle are referred to as ‘surplus’ to treatment needs, or ‘spare’.

Spare embryos (unused on day 2 or 3 of cleavage) may be kept in culture for two or three more days to the blastocyst stage, and/or cryopreserved for a later ‘frozen embryo transfer’ (see below for more detail). Alternatively, they may be discarded, donated for another woman’s/couple’s treatment, or donated to research, including hESC research.Embryos that score low on appearance and progress are classed as poor quality and are usually ‘left to perish’ and then discarded or donated for (non hESC) research. Occasionally (e.g., in the absence of top quality embryos) poor quality embryos are transferred even though they seem unlikely to develop into a pregnancy, because it is very hard to be *certain* they have no chance of success.

Uncertainty is difficult for staff because patients look to staff for guidance and recommendations about how many or which embryos to transfer, freeze, discard or donate. For our purposes here, we focus less on the implications of uncertainty for individual staff members and more on the incorporation of social and ethical values in *grading practices* as a response.

### Fluidity in grading criteria to give embryos ‘every chance’

Tensions between fluidity and stablisation were evident in responses from staff to uncertainty in classifying embryos and we discuss these in this and the following section in turn.

One area of concern voiced by staff about uncertainty in grading embryos related to the idea of embryos having ‘chances’, or giving them ‘every chance’, which had a number of meanings. For example, they described the attrition of chances in terms of fertilisation after egg retrieval:

‘… on the whole every egg gets its chance to be fertilised. And next day, those that haven’t fertilised have had their chance and if they’re not fertilised, they’re discarded or go to research.’ (Nurse 1)

Similarly, some staff responded to uncertainty about which embryos have the most promise by framing this attrition as embryos ‘deciding for themselves’. If embryos were not successful, they could at least be seen as having had their ‘chance’.

Giving embryos every chance could also mean deciding to transfer poorer quality embryos because women/couples did not want or could not afford to have another cycle of treatment, and a relatively poor embryo was the ‘best’ one in a cycle. In these cases some staff thought it was kinder to give an embryo ‘every chance’ (and give the woman/couple every chance to become pregnant), but carefully explained that success was not likely. Further, it could mean making sure that at every step in the treatment, embryos were given the chance of staying on the ‘pregnancy trail’ as long as possible, reflecting a strongly held value amongst staff of ‘patient-centredness’, i.e., commitment to the patient’s treatment goal of pregnancy.

Many accounts were given of embryos graded as poor that were nevertheless transferred and proved successful. This gave the impression that both clinical and embryology staff felt ambivalent about relying too strictly on the grading process. Those cases had made staff wonder if some embryos that were not ‘given a chance’ could have created a baby, suggesting the danger of a form of unintentional harm. Fluidity in classifying embryos as worth transferring therefore seemed to some staff a necessary feature of a fair grading system. Fluidity takes into account factors such as giving patients hope, or ideas about fairness, avoiding harm, and giving embryos a chance, illustrating social and ethical aspects of embryo classifying work.

### Fairness and standardisation

An alternative response to uncertainty and possible unfairness was that it seemed to some staff kinder to women/couples to be stringent in classifying embryos according to a more fixed standard (i.e., only allowing top quality embryos to be transferred or frozen) so as not to give women/couples false hopes and to achieve another kind of fairness (i.e., tying the chances for each embryo more strictly to their morphology).

Embryologists and clinicians explained that grading embryos is not an exact science, acknowledging variations between clinics and to some extent within clinics. In all three clinics, inter-reliability between embryologists was attempted. For example, embryologists consulted each other regularly to compare their grading of embryos, and in two of the sites, embryologists independently classified a sample of embryos in photographs to compare scores. Some implications of grading embryos too stringently were voiced by Embryologist 45:

‘I am quite relaxed with how I treat my embryos, I will give them all the chance that I possibly can without breaking the rules! …we are quite different in, 1) how we grade, and 2) what we consider for freezing and for transfer. Some people are quite harsh and will say, ‘No it’s not good enough,’ whereas other people are, you know, ‘I’m not sure, therefore I will give it the benefit of the doubt.’ And I personally think that’s really unfair, because the patient will get different treatments depending on who is looking at their blastocyst at that particular time.’

This embryologist justified her ‘relaxed’ approach to ‘the rules’ in terms of giving embryos every chance. At the same time, she saw variation in grading as ‘really unfair’. She seemed to advocate being less ‘harsh’ by allowing embryos ‘the benefit of the doubt’ and to imply that others should adopt this line so that patients are given the same ‘relaxed’ approach to treatment.

The UK Association of Clinical Embryologists recently proposed a national standard grading system to overcome differences in grading and freezing criteria, to be introduced in 2010 (Cutting, Morroll, Roberts, Pickering, & Rutherford, 2008). Embryologist 2 thought that any national standard grading system

‘should allow for differences in criteria, because every clinic does have differences in, for example, the embryo grading criteria. And the number of embryos that, you know, they are freezing.’

Further, she believed that although grading criteria might differ, patients were not ‘worse off’ in different clinics as a result. On the contrary:

‘I think it’s fine for patients to go to all the clinics who deal with it slightly differently and I think that might be good for them, in a way that they may actually be better off trying something, you know, a different technique in a different clinic.’

These quotes illustrate from slightly different perspectives how ideas of fairness and patient benefit play a part in constructing or adhering to a standardised grading system. A fair system did not necessarily result from grading strictly to a uniform standard, but fluidity could be used to incorporate social and ethical values such as equity and giving embryos every chance.

### Freezing embryos extends choice, control and chances, and prevents regret

Classification of embryos as suitable for *freezing* also has important social and ethical implications. Many of the staff thought that freezing embryos gives patients more choices and control because it introduces further opportunities to use embryos than is possible in the fresh cycle, and that donating frozen embryos is *preferable* to donating good quality fresh embryos to research because it reduces a form of uncertainty and possible regret. As Counsellor 6 explains,

‘…at that point in time [during the fresh cycle] they don’t know. [Donating fresh embryos] is relinquishing chances because they don’t know whether the embryos that they are using are going to create a pregnancy or not. So it’s much more like, well, parallels with egg sharing, because if I give half my eggs away, those might be the good eggs. Mine might not be so good.’

Similarly, Embryologist 5 said:

‘I can’t imagine anyone who would be happy that they had donated half their embryos for research and not been able to get pregnant.’

If a ‘spare’ *fresh* embryo were donated for research, women/couples could not be sure *at that point* that they were not ‘giving away’ a chance of pregnancy, which supports McLeod & Baylis (2007). Staff from both the ACU and stem cell laboratories felt more ‘comfortable’ asking women/couples to donate frozen embryos when their treatment was finished, they had had children, or decided they no longer wanted to use the embryos. Stem Cell Scientist 9 commented,

‘I prefer cryopreserved [embryos] because … it’s at the end of their treatment and they’ve forgotten about them. They’re more or less, ‘That’s it, I don’t want any more to do with the embryos, but they might as well be used for research … the process of making stem cells destroys the embryo and otherwise they would be destroyed, therefore why not use them for that purpose?’

Clinician 35 agreed with the proposal in one of the EDGs that freezing is morally and psychologically helpful to women/couples because it gives them a period of adjustment:

‘Yes. Especially because then they know that absolutely every embryo that could possibly have potential has been frozen for their use, and that research is a secondary option, once they’ve used whatever they want for themselves... It’s totally different to taking embryos out of a fresh cycle and you say, ‘These aren’t good enough for you, we’ll have them.’

So freezing embryos transforms them into a morally preferable class of ‘spare’ embryo, because women/couples who do not use them for treatment have had more time to come to a decision to donate them to research, and to know that their embryos have had every chance to remain on the ‘pregnancy trail’.

Not all ‘spare’ embryos are frozen however. Embryologist 2 explained:

‘if there’s any spare that aren’t high enough quality to freeze or transfer, then we would obviously say to the patient, ‘Do you want these to go to research?’

This quote refers in an almost taken for granted way to the further assessment of embryos when classifying ‘spare’ embryos as ‘high enough quality to freeze or transfer’. Some clinics select the best one or two fresh embryos to transfer, but don’t recommend freezing if there are only one or two good quality embryos left, which makes those embryos ‘spare’ even though they may be top quality (Haimes & Taylor, 2009). This was justified at the time on grounds of survival risks after thawing but also on cost and not giving patients false hopes, illustrating further how the constitution of ‘spare’ embryos includes more than ‘purely technical’ contingency.

Another example of the constantly shifting *policy* environment for clinical decisions about freezing embryos is the elective single embryo transfer policy (eSET). Following clinical and technical innovation, this policy was adopted at the national level in January 2009 to reduce the multiple birthrate after IVF, (Braude, 2006). It will have important impacts on freezing decisions, particularly in clinics that have a minimum number to freeze policy. As Embryologist 5 explains:

‘…we say three mainly if a patient is having two embryos back [i.e. two transferred, and three frozen]. But now we’re moving on to put just one embryo back, we are saying to patients, ‘We will freeze just two embryos for you.’

This policy will have the greatest impact where patients are not funded by primary care trusts (PCTs) for any treatment or for freezing specifically (the majority). As Embryologist 7 argued:

‘Elective single embryo transfer will not work in this country unless the PCTs fund frozen cycles’.

If funding does not increase, either the policy will fail (with all the cost implications to the NHS that follow), or patients will be put in the arguably unfair position of being asked to transfer only one fresh embryo even if they cannot afford to freeze any others in that cycle for a future transfer, creating another category of good quality ‘spare’ embryos taken off the ‘pregnancy trail’ for social rather than scientific reasons. According to this view, adequate funding is essential to a ‘fair’ eSET grading policy.

### Ethical preference for greater certainty from extended culturing

Until recently, most UK clinics identified suitable embryos for transfer and freezing at the cleavage stage (on day two or three post egg collection). At the time of writing (autumn 2009), for couples who have a sufficient number of high quality embryos at the cleavage stage, an increasing number of clinics now offer a further period of in-vitro culturing up to the blastocyst stage (day five or six). This gives embryologists more time to assess embryo development as part of the grading system is assessment of developmental progression.There is some evidence that frozen blastocysts have a significantly higher thawing success rate (e.g., 90% compared to 60–70% for cleavage stage embryos) and that “where equal numbers of good quality embryos are available, the transfer of a single blastocyst on day five shows a significantly higher pregnancy and live birth rate compared to transfer of a single embryo on day three” (Cutting et al., 2008, p. 138; Papanikolaou et al., 2008).

Keeping embryos on the ‘pregnancy trail’ by culturing them on to the blastocyst stage and freezing illustrates both attrition and extending of ‘chances’:

‘…it’s a steeplechase – they’ll all get over the first fence, a few will get over the second…the longer you leave them, the fewer you have that are continuing to develop. And so then you can be more certain they’re the ones that have the best potential. So we don’t do any freezing now on the third day, even if we put the embryos back on that day. If we were thinking about freezing, we would keep the embryos another couple of days, until the fifth day. If they then make it to good quality blastocyst, then we would freeze them. … if they have the potential, they will be able to show that potential… we don’t want to freeze embryos that don’t have that potential to carry on developing. (Embryologist 34)

In this quote the ‘steeplechase’ gives embryos more time to ‘show that potential’ by culturing them for another two days. It also means fewer embryos may be frozen in that cycle. The argument is that only ‘better’ blastocysts are frozen, with their better chances of success, rather than the less certain cleavage stage embryos, and again includes ideas about giving people false hopes.

Changing to blastocyst culturing in clinics with a ‘minimum number to freeze policy’ could make a substantial difference to the number of embryos that women/couples could have cryopreserved. It allows clinics to be more confident of the success of freezing a single blastocyst, but has the consequence that fewer good quality fresh cleavage stage embryos would be available for freezing or for research.

Staff in the unit with the most developed blastocyst programme claimed it is less ethical to approach couples for cleavage stage embryos than blastocysts for research, because blastocyst culturing provides more information on which to make grading decisions. Their concern was that if a cleavage stage embryo donated for research went on to develop successfully into a blastocyst, which is a prerequisite for starting hESC derivation, it could seem that the clinic had failed to make the right assessment, and the couple would be deprived of a possible ‘chance’ for pregnancy. By extension, no *fresh* day three embryo should be donated to research (with some exceptions as noted above), *in case* it looked better by day five in the hESC laboratory. As Embryologist 34 explained:

‘any embryo that has the capacity to make an inner cell mass and to make a stem cell line, should be kept for the patient. So it’s a mistake really if you have any embryos that do that, but haven’t been used for the patient. That’s a mistake in your grading system.’

Later on in the interview, she returns to thinking through what troubles her about this situation:

‘… the missing link in some ways is… nobody really sort of says about the fact that embryos for research are good quality embryos that potentially do have that, well that have that potential to make a pregnancy.’

In this case, the ‘dual reproductive value’ of embryos is troubling, and moving embryos onto the ‘research trail’ too soon could be deemed a grading ‘mistake’. Keeping embryos on the ‘pregnancy trail’ for the maximum time meant the unit could avoid the chance of missing an embryo’s potential at day three. The implications of such possible ‘mistakes’ were troubling, again, not only in a ‘technical’ sense. Blastocyst culturing and freezing constituted a defense against the possibility of couples donating embryos to research that could have produced a baby.

### Naturalisation of frozen and PGD affected embryo categories as ethically acceptable

So far, we have argued that many staff prefer to retain fluidity in embryo classifying work to ameliorate uncertainty and accommodate technical, social, ethical and policy contingencies. Notwithstanding this fluidity, developments in cryopreservation, blastocyst culturing, and the eSET policy have made discarding or donating good quality fresh embryos to research arguably less justifiable than using frozen ones. The ‘spareness’, or ethical acceptance of using *frozen* embryos for research that couples no longer want, has become more fixed or ‘naturalised’ (Bowker & Star, 1999). The troubling contingencies and many of the uncertainties inherent in grading fresh embryos fall away, the meaning of the object in this social world becomes taken for granted, and the community of practice relates to it in a more routine way.

The concept of naturalisation applies well to a further category: ‘affected PGD embryos’. Staff in a clinic with an advanced PGD programme argued for the ethical acceptability of asking women/couples to donate ‘affected PGD embryos’ as an ethically distinct kind of *fresh* spare embryo. These embryos may be viable or even excellent quality, but are not used for the couple’s treatment once PGD has identified them as affected by the genetic condition they are hoping to avoid in their potential child. Clinician 35 was happy for embryos to be donated for research from two categories:

‘…if you were in an ideal world and there were enough embryos to research, *either through PGD or from frozen cycles*, that would be great.’

Affected PGD embryos were thought to be logically fair to recruit for research, because PGD patients had already decided that they would not want to use them as part of their treatment. Another reason was that staff imagined that PGD patients have benefited from research themselves and so might be more inclined to think in terms of ‘giving something back’:

‘I suppose if I try to put myself in that position, then I would feel like somehow I could give something back… using the technology to have the embryos that I want, and the ones that I don’t want, I think I will be quite happy to give those to research… that’s the ones I’m happiest, in a lab, to give to research, because I know the patient doesn’t want them.’ (Embryologist 34)

Going further, in one EDG, Stem Cell Scientist 35 shared the frustration voiced in the group that some PGD patients choose to destroy embryos rather than allow them to be used for research:

‘It’s a waste. When they have only been able to get that far by using other people’s embryos that have gone to research.’

Even though affected PGD *embryos* were seen as more acceptable to use for research, Nurse 40 did not want PGD *patients* to be taken for granted as donors to research. She was concerned that they could:

‘become an easy target because… they know about their condition, they know what they’ve gone through, they know that this kind of treatment will certainly help others in future, and it’s much easier to approach them because you know they will understand. But at the same time… they might be some people with strong religious or cultural backgrounds that sort of hinders them to, that they wouldn’t be happy to donate. So I think… they should still understand that it is their choice.’

This quote illustrates a danger in all classification systems but particularly where categories become naturalised. Ideas about the obligation of particular kinds of patients extrapolated from a specific category of ‘spare’ embryo could override other social and ethical values for these patients. The reasons for the formation of such categories and their moral content can become taken for granted, and some values may no longer be articulated or visible.

The naturalisation of affected PGD embryos as a morally accepted category of fresh embryo for donation to hESC research illustrates Bowker and Star’s (1999) point that classification systems have the potential to privilege particular values, which may be expressed in the built moral environment. In this case, this naturalisation has facilitated the fulfilment of architectural efforts to support the IVF-stem cell interface.

## Discussion

Harking back to the reference in our introduction to Bowker and Star’s (1999) characterisation of interrogating infrastructures as similar to how city planners or historians could analyse highway permits and zoning decisions to tell a city’s story, our aim here has been to analyse the classification of embryos to tell a story about the building of linked ACU and stem cell laboratories at a particular time and in a particular place. Investment of substantial funds in new connected ACU and GMP stem cell laboratories was part of the political commitment to exploiting the ‘dual reproductive value’ of IVF embryos for the development of stem cell science in the UK. At the time of writing, however, and with the important exception of ‘affected PGD embryos’, the practice of discarding good quality *fresh* embryos or donating them to research (necessitating the architectural link) is seen as less ethically acceptable in the clinics we studied now that blastocyst culturing and more successful freezing techniques have extended the time embryos can remain on the ‘pregnancy trail’.

Extended culturing and freezing techniques have enhanced the ability of staff to privilege the ethical value of ‘giving embryos every chance’ to continue on the treatment trajectory more reliably and for longer than was normally possible only a few years ago. Before these developments, the donation of fresh embryos was thought to be more justifiable in the clinics participating in our study. Our observation that the stem cell laboratories that participated in our research are predominantly using frozen (rather than fresh) IVF embryos brings into question whether investment in and architectural manifestations of the ‘IVF-stem cell interface’, have so far been as productive in respect to using fresh embryos as was envisaged.

As Bowker and Star (1999) argue, classification systems, the work they do, and contested categories (Bauer & Wahlberg, 2009) are socially and politically charged. This theoretical position has been applied by others interested in the ‘messiness’ of classification in the laboratory and clinic (e.g., Latimer et al., 2006; Hedgecoe, 2003) and in standardisation (Kerr, 2008). While these writers examine contingency in the accomplishment of clinical categories, ethical issues in the use of classification, and social and ethical *consequences* of clinical and scientific practices, we have observed the incorporation of moral considerations within the classification construction process itself. This may be equally contingent and ‘messy’, but the important point here is that moral contingencies are part of the formation of these categories rather than classifications having moral consequences after being formed.

This builds on our work in an earlier project on classification of PGD embryos in which we argued that PGD embryos could be understood as ‘moral work objects’ (Ehrich et al., 2008). In the PGD study we were more concerned with choices that were based on women’s/couples’ wish to avoid having children who were affected by specific genetic conditions. In this study we have looked more closely at how embryos are graded in IVF rather than in PGD, i.e., how IVF embryos are assessed as not suitable for transfer in the treatment cycle, rather than (as with PGD embryos) because they are affected by a genetic condition. The production of the category of ‘spare’ embryos as illustrated in the two studies is important for different reasons and raises different questions. Our analysis of these issues in this study was aided by drawing on the emphasis in Bowker and Star’s (1999) work on how classification systems not only produce categories such as ‘spare’ embryos but also how they can produce forms of infrastructure, in this case the built environment of linked ACU and stem cell laboratories.

Our aim in this paper was not to make policy recommendations but to illuminate the subtle and nuanced ethical issues that are imbricated with technological and policy contingencies in this classifying work. Some of the policy implications arising from our data are addressed in a forthcoming paper on the possibility of enabling embryo donors to restrict the purposes to which scientists might use hESC lines that are derived from their embryos. The single embryo transfer policy is addressed in more detail in a further forthcoming paper exploring legal aspects of consent to donation of embryos to research, including the issue of how much control women/couples can have over freezing decisions.

In this paper we have illustrated how the category of ‘spare embryo’ can be seen as having been constituted as a moral work object in both fluid and naturalised forms. On the one hand, it has resisted becoming a fixed, taken for granted, category, because of changing technological but also other contingencies in its constitution as a moral work object. Ever-changing technical contingencies, as well as ethical uncertainties in decisions about the fate of assisted conception and PGD embryos, point to the *fluid* constitution of embryos as particular kinds of moral work objects.

On the other hand, the categories of ‘frozen’ and ‘affected PGD’ embryos provide interesting and compelling exceptions to this relative fluidity in categories and appear to have become more fixed, or ‘naturalised’, at least in the UK (e.g., compared to Denmark (Svendsen & Koch, 2008, p. 106)). Where ‘affected PGD embryos’ can be donated directly for hESC research, their dual reproductive value, and the purpose of the new buildings to facilitate the IVF-stem cell interface, may be more likely to be fulfilled. Put another way, this ‘built moral environment’ has had more success where at least one category of ‘fresh spare embryo’ (i.e., ‘affected PGD’) has become naturalised or fixed, and less where the ethical status of fresh embryos remains more contingent and fluid.

New embryo categories and work practices emerge and mutually constitute each other, and particular categories, such as fresh or frozen ‘spare’ embryos, and ‘affected PGD embryos’, are infused with technical, clinical, social and ethical meanings and values. Yet we observe that the moral content of such categories is often taken for granted or less ‘visible’. We argue here that articulation of the social and moral meanings and values inherent in emergent categories is of equal importance to a focus on technical aspects, as illustrated by our case study of the substantial implications of this mutual constitution for the development of new technologies and the ‘built moral environment’.

To conclude, we have tried to make social and ethical elements of professional classification practices at the ‘IVF-stem cell interface’ more visible, and at the same time to illustrate the importance of understanding the detailed development of these technologies in order to trace changes in ethical and moral aspects of their use in human reproduction treatments. The success of the building projects planned to facilitate the IVF-stem cell interface will depend not only on technological solutions provided by the material architecture, but on an understanding of the imbrications of social, ethical, technological and clinical contingencies, to produce the ‘built moral environment’, and embryos as particular kinds of moral work object.
